# In Vivo Imaging Enables High Resolution Preclinical Trials on Patients’ Leukemia Cells Growing in Mice

**DOI:** 10.1371/journal.pone.0052798

**Published:** 2012-12-31

**Authors:** Nadia Terziyska, Catarina Castro Alves, Volker Groiss, Katja Schneider, Katarina Farkasova, Manfred Ogris, Ernst Wagner, Harald Ehrhardt, Renier J. Brentjens, Udo zur Stadt, Martin Horstmann, Leticia Quintanilla-Martinez, Irmela Jeremias

**Affiliations:** 1 Department of Gene Vectors, Helmholtz Zentrum München, German Research Center for Environmental Health, Munich, Germany; 2 Pharmaceutical Biotechnology, Center for System-Based Drug Research, Department of Pharmacy, Ludwig-Maximilians-Universität, Munich, Germany; 3 Department of Medicine and the Center for Cell Engineering, Memorial Sloan Kettering Cancer Center, New York, New York, United States of America; 4 Research Institute Children’s Cancer Center, Paediatric Haematology and Oncology and Center for Diagnostic, University Medical Center Hamburg Eppendorf, Hamburg Eppendorf, Germany; 5 Institute of Pathology, Eberhard-Karls-Universität Tübingen and Comprehensive Cancer Center, University Hospital, Tübingen, Germany; 6 Department of Oncology, Dr. von Haunersches Kinderspital, Ludwig Maximilians University, Munich, Germany; B.C. Cancer Agency, Canada

## Abstract

**Background:**

Xenograft mouse models represent helpful tools for preclinical studies on human tumors. For modeling the complexity of the human disease, primary tumor cells are by far superior to established cell lines. As qualified exemplary model, patients’ acute lymphoblastic leukemia cells reliably engraft in mice inducing orthotopic disseminated leukemia closely resembling the disease in men. Unfortunately, disease monitoring of acute lymphoblastic leukemia in mice is hampered by lack of a suitable readout parameter.

**Design and Methods:**

Patients’ acute lymphoblastic leukemia cells were lentivirally transduced to express the membrane-bound form of Gaussia luciferase. In vivo imaging was established in individual patients’ leukemias and extensively validated.

**Results:**

Bioluminescence in vivo imaging enabled reliable and continuous follow-up of individual mice. Light emission strictly correlated to post mortem quantification of leukemic burden and revealed a logarithmic, time and cell number dependent growth pattern. Imaging conveniently quantified frequencies of leukemia initiating cells in limiting dilution transplantation assays. Upon detecting a single leukemia cell within more than 10,000 bone marrow cells, imaging enabled monitoring minimal residual disease, time to tumor re-growth and relapse. Imaging quantified therapy effects precisely and with low variances, discriminating treatment failure from partial and complete responses.

**Conclusions:**

For the first time, we characterized in detail how in vivo imaging reforms preclinical studies on patient-derived tumors upon increasing monitoring resolution. In the future, in vivo imaging will enable performing precise preclinical studies on a broad range of highly demanding clinical challenges, such as treatment failure, resistance in leukemia initiating cells, minimal residual disease and relapse.

## Introduction

Preclinical mouse models are helpful tools for studying biology and therapy of diseases. Novel therapeutic approaches undergo detailed preclinical evaluation before translation into clinical trials [1]. In the present work, a defined preclinical leukemia mouse model was technically improved to allow decisive studies on clinically demanding challenges.

In cancer research, a variety of different mouse models exist including xenotransplantation models and syngeneic models [2]. Xenotransplantation models enable studying human tumor cells upon growth in severely immunocompromised mice [3]. Within the xenotransplantation models, the use of primary patients’ tumor cells is superior to the use of cell lines, as primary cells enable modeling of the complex heterogeneity of human tumors, while cell lines might have acquired non-physiologic mutations upon prolonged culture in vitro [4].

At best, xenotransplanted tumor cells generate a disease in mice which highly resembles the disease in men [5]. Nevertheless, transplantation of solid tumors might suffer from heterotopic tumor localization and metastasis in mice [5]. In contrast, tumor cells obtained from patients with acute lymphoblastic leukemia (ALL) engraft and develop the disease in mice with an organ distribution highly similar between mice and men [6]. In fact, since 2 decades the xenotransplantation model of patient ALL cells is well characterized [7] and fulfills many criteria requested for preclinical treatment trials. Due to high engraftment rates, the heterogeneity of ALL can be modeled in mice and trials are performed in genetically defined subgroups of ALLs [6]. Nevertheless, first engraftment in mice might be non-representative for the heterogeneity of the human sample [8] and clonal evolution might take place upon passaging cells through mice, although reportedly at a minor level [9]. Taken together, xenotransplantation of primary human ALL into mice emerges as attractive model for preclinical anti-cancer trials in general.

Nevertheless, sensitive follow up of leukemia progression in mice remains a limitation of the model. Invasive bone marrow aspirations in mice require prolonged periods of recuperation; blood sampling is hampered by late and heterogeneous presence of tumor cells into the peripheral blood [10]. Lack of sensitive and convenient follow up of the leukemic disease so far disabled quantifying treatment responses and differentiating distinct clinical disease stages.

In vivo imaging based on molecular cell marking represents a sensitive readout parameter to monitor xenotransplanted tumors in mice, e.g., using bioluminescence [11], [12]. So far, in vivo imaging was mainly performed in preclinical models using tumor cell lines as patient-derived tumor cells are more difficult in handling, e.g., for molecular manipulation. Patient-derived tumor cells do not grow in vitro; instead, they survive only few hours in culture.

Here, we established the molecular labeling of patient-derived ALL cells and characterized in detail, how bioluminescence in vivo imaging enables a novel level of precision for future preclinical studies. In vivo imaging enabled quantification of treatment effects and monitoring of minimal residual disease in mice. The improved mouse model will allow performing decisive and complex preclinical studies on individual leukemias in the future.

## Design and Methods

### Ethical Statements

Written informed consent was obtained from all patients and from parents/carers in the cases where patients were minors. The study was performed in accordance with the ethical standards of the responsible committee on human experimentation (written approval by Ethikkommission des Klinikums der Ludwig- Maximilians-Universität München, Ethikkommission@med.uni-muenchen.de, April 15/2008, number 068-08) and with the Helsinki Declaration of 1975, as revised in 2000.

All animal trials were performed in accordance with the current ethical standards of the official committee on animal experimentation (written approval by Regierung von Oberbayern, poststelle@reg-ob.bayern.de, May 10/2007, number 55.2-1-54-2531-2-07).

### Cloning and Production of Lentiviruses

The GLuc construct encoding for the human CD8 leader peptide and the CD8 transmembrane domain fused to GLuc [13] was subcloned into the multicloning site of pCDH-EF1-MCS-T2A-copGFP vector (System Biosciences, Mountain View, CA, USA) using EcoRI and BamHI. The 3′ stop codon was removed during PCR amplification.

The third generation packaging plasmids pMDLg/pRRE, pRSV-Rev and pMD2-G [14] were kindly provided by T. Schroeder. High-titer vesicular stomatitis virus (VSV) G protein-pseudotyped lentivector was prepared by transient four-plasmid transfection of 293T cells using Trans-IT®-293 Transfection Reagent (Mirus, Madison, WI, USA) and supernatant concentration as described [15]. The functional titer of virus was determined by infection of 293T cells with serial dilutions of the vector stock, followed by cytometric analysis of GFP positive cells. Viral titer was set to 5×10^8^ transduction units/ml.

### Generation of GLuc Expressing Patient-derived ALL Cells

Patient-derived leukemia cells were freshly isolated from mouse spleens, purified and cultured in RPMI medium supplemented with 20% FCS, 1% penicillin/streptomycin, 1% gentamycin, 6 µl/ml mixture of insulin, transferrin and selenium (Invitrogen, Carlsbad, CA), 1 mM sodium pyruvate and 50 µM 1-thioglycerole (Sigma-Aldrich, St. Louis, MO) in the absence of further cytokines as described [16], [17]. Cells were transduced overnight with GLuc virus in the additional presence of 3 µg/ml polybrene (Sigma, Hamburg, Germany). After extensive washing in phosphate-buffered saline (PBS) plus 2% FCS to remove vivid virus, 1–3 million cells per mouse were injected intravenously into recipient NSG (NOD-scid IL2Rgammanull, The Jackson Laboratory, Bar Harbour, ME, USA) mice for amplification. After passaging, cells were sorted using the FACSVantage SE machine (BD Biosciences) and re-amplified in mice. For samples ALL-4S and ALL-50 cell sorting was repeated once after re-passaging through mice.

### Bioluminescence in vivo Imaging

The IVIS Lumina II Imaging System was used (Caliper Life Sciences, Mainz, Germany). Mice were anesthetized using isoflurane, placed into the imaging chamber in a supine position and fixed at the lower limbs and by the inhalation tube. Coelenterazine (Synchem OHG, Felsberg/Altenburg, Germany) was dissolved in acidified methanol (HPLC grade) at concentration 10 mg/ml and diluted shortly before injection in sterile HBG buffer (HEPES-buffered Glucose containing 20 mM HEPES at pH 7.1, 5% glucose w/v). Immediately after intravenous tail vein injection of 100 µg of native Coelenterazine, mice were imaged for 15 seconds using a field of view of 12,5 cm with binning 8, f/stop 1 and open filter setting. To monitor tumor growth, mice were imaged once weekly; after therapy, mice were imaged every other day.

### Quantification of Imaging Pictures

The Living Image software 4.x (Caliper Life Sciences, Mainz, Germany) was used for data acquisition and quantification of light emission using a scale with a minimum of 1,8×10^4^ photons per second per cm^2^ per solid angle of one steradian (sr). Different regions of interest (ROI) were defined and signals were considered positive, when light emission exceeded background in each ROI; background was measured in 15 mice harboring GLuc negative leukemias; a ROI covering the entire animal was used (background 4×10^6^ photons per second); as an exception and to determine early engraftment = minimal disease, a small ROI (0,35 cm^2^; background 6×10^4^ photons per second) was set at femurs at the location, where and when first light emission became visible; depending on the expression level of the transgenes, overt leukemia was considered above 10^9^–10^10^ photons per second using the ROI covering the entire animal; overt leukemia served as criterion for ending experiments, as it shortly preceded onset of clinical signs of disease in mice.

### Preclinical in vivo Treatment Trials

Control animals received physiological salt solution intraperitoneally; treatment group mice were injected i.p. with a single dose of either Etoposid (VP-16; 50 mg/kg; Sigma, Hamburg, Germany) or Cyclophosphamide (Cyclo; 150 mg/kg; Baxter, Unterschleissheim, Germany) diluted in 0.9% NaCl.

### Statistical Analysis

Mean and standard error of the mean (SEM) were calculated using the Microsoft Excel 2010 software (Microsoft, Redmont, WA, USA). To determine significance of treatment effects in vivo, Mann-Whitney Rank Sum Test and the Sigma Plot 12 software (Systat Software, Erkrath, Germany) was used. CSC frequencies were calculated according to Poisson statistics using the ELDA software application (http://bioinf.wehi.edu.au/software/elda).

### Additional Methods

See Supporting Information S1 for description on the animal model, LDTA, qRT-PCR, flow cytometry, histology and in vitro apoptosis assays.

## Results

The aim was to introduce and validate in vivo imaging as novel readout parameter for monitoring patient-derived leukemias growing in mice. Sensitive and reliable disease monitoring will allow decisive preclinical studies on a novel level of complexity and accuracy in the future.

### Generation of Gaussia Luciferase-expressing Patient-derived ALL Cells

To establish in vivo imaging as readout for monitoring individual ALLs in mice, Gaussia luciferase (GLuc) was chosen in its membrane-bound form [13]. GLuc emits highly intensive light compared to luciferases of other species and is especially useful in monitoring superficial organs such as bone marrow [18].

**Figure 1:**
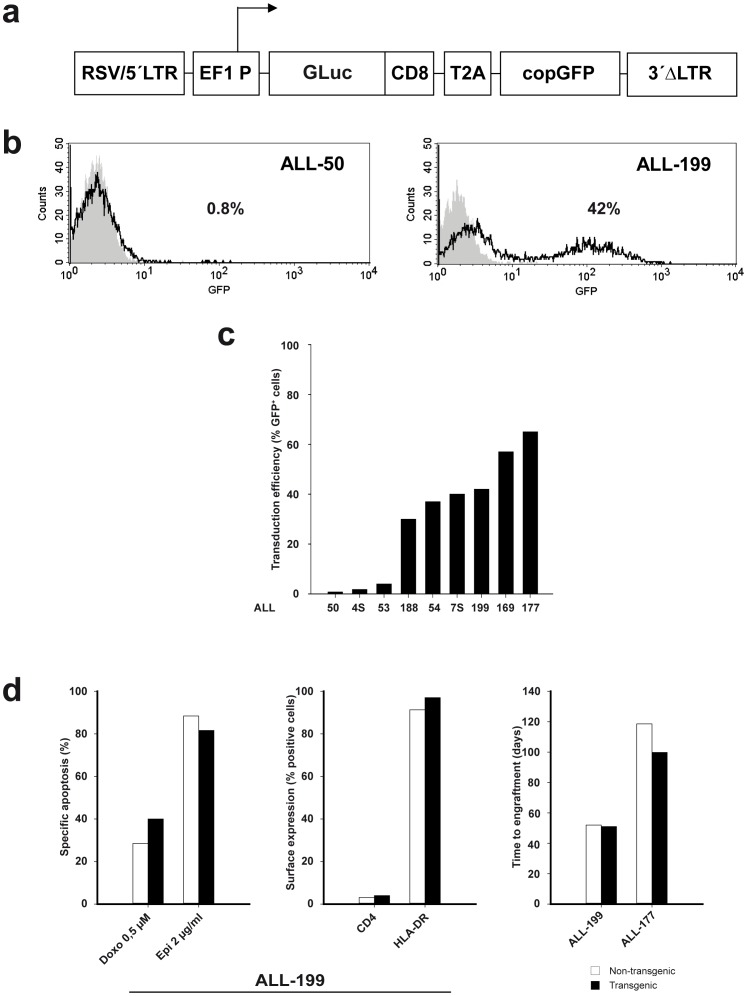
Generation of GLuc **-expressing patient-derived ALL cells. a** Scheme of the lentiviral vector construct; arrow indicates start of transcription; RSV/5′LTR = hybrid of the Rous Sarcoma virus promoter and the U5 long terminal repeat from HIV-1 virus; EF1 P = constitutive elongation factor 1-alpha promoter; GLuc = membrane anchored form of the Gaussia luciferase (GLuc) enzyme fused to the transmembrane domain of CD8; T2A = “self-cleaving” 2A peptide from insect virus *Thosea asigna*; copGFP = green fluorescent protein cloned from copepod *Pontellina plumata*; 3′ΔLTR = HIV-1 virus long terminal repeat with a self-inactivating U3 deletion; **b, c**Transduction efficiency as determined by flow cytometry measurement of GFP expression after one round of amplification of transduced cells in mice; (**b**) in ALL-50 and ALL-199; (**c**) in all 9 patient-derived ALL samples studied; **d** Stability of biological characteristics of patient-derived ALL cells despite of lentiviral transduction; examples from data shown in detail in Supplemental Figure 1; comparison of ALL-199 cells before and after lentiviral transduction and sorting concerning drug-induced cell death after 48 hours in vitro (left panel), expression of cell surface markers (middle panel) and time to engraftment (right panel).

Leukemia cells are notoriously difficult to transfect and patient-derived leukemia cells do not allow antibiotics-based selection in vitro. Therefore, lentiviral transduction was chosen, although transgene integration into unsuitable genomic sites might alter cell function. GLuc was cloned into a lentiviral vector harboring additionally copepod green fluorescence protein (GFP) (Figure 1a). Primary ALL cells were passaged through mice at least once. Cells freshly isolated from mouse spleens were transduced overnight using a multitude of infection of 30–100 lentiviruses per cell in the presence of 3 µg/ml polybrene without further addition of cytokines. Next morning and after extensive washing in phosphate-buffered saline (PBS) plus 2% FCS to remove vivid virus, 1–3 million cells per mouse were injected intravenously into recipient NSG (NOD-scid IL2Rgammanull, The Jackson Laboratory, Bar Harbour, ME, USA) mice for amplification. After passaging, cells were sorted using the FACSVantage SE machine (BD Biosciences) and re-amplified in mice. For samples ALL-4S and ALL-50 cell sorting was repeated once after re-passaging through mice.

All 9 samples from children with ALL (1 T-ALL and 8 B-ALL, clinical data in Supplemental Table 1) were successfully transduced, although transduction efficiencies and levels of transgene expression varied widely between samples (Figure 1b and 1c). While high transduction rates ensured molecular staining of representative cells, low transduction rates contained the risk of selecting non-representative cells. Transduced cells were enriched by 1 or 2 rounds of cell sorting using GFP to above 90% in all samples (data not shown); transgene expression remained stable over passaging suggesting successful transduction of leukemia initiating cells (Supplemental Table 2). Transduction and expression of transgenes did not alter important functional biological characteristics of patient-derived ALL cells, not even after various rounds of passaging through mice (Figure 1d, Supplemental Figure 1). For details please refer to the suppl. Results section. Taken together, lentiviral transduction enabled generating patient-derived ALL cells expressing transgenes without altering the described basic biological cell characteristics studied. In the future and using transgenes other than marker genes, the technique will enable molecular signaling studies in patient-derived leukemia cells.

### In vivo Imaging of Patient-derived ALL in Mice

For imaging, the convenient IVIS Lumina II Imaging System (Caliper Life Sciences) was used together with an optimized protocol (for details see suppl. Results). Kinetics of GLuc-emitted light from leukemia cells was similar to published kinetics on GLuc-expressing T-cells [13] (Supplemental Figure 2). Injection of Coelenterazine resulted in a substrate-related light emission from the liver independently from the presence of GLuc in all mice; in non-leukemic control mice (data not shown), in mice bearing a non-transgenic leukemia (Supplemental Figure 3a) or in mice bearing leukemia transgenic only for expression of GFP, but not GLuc (Supplemental Figure 3b). The unspecific liver signal did not interfere with evaluation of the leukemic disease as it was of minor intensity. The imaging procedure was performed easily in handling and well tolerated by all mice with a nearly absent imaging-related death rate restricted to mice with highly advanced leukemia.

**Figure 2:**
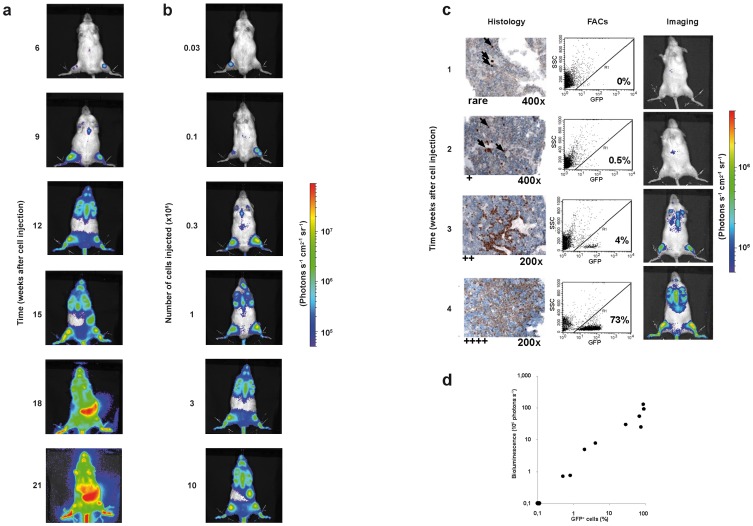
In vivo imaging of patient-derived ALL in mice. a Kinetics of leukemic growth; 5×10^4^ ALL-177 cells per mouse were injected into a group of 5 mice which were imaged repeatedly over time; a single representative mouse is shown; units in rainbow color scales are photons per second per cm2 per steradian (photons s^−1^ cm^2−1^ sr^−1^); **b** Dose-response of leukemic growth; 1×10^7^–3×10^4^ serially diluted ALL-177 cells were injected into groups of 5 mice which were imaged 8 weeks after injection; a single representative mouse is shown for each group; **c, d**Good correlation of in vivo imaging to post mortem readout parameters; 12 mice were injected with 10^5^ ALL-50 cells/mouse; each week, 2 mice were imaged and sacrificed; organs were collected and cell suspensions prepared from half the organ and 1 femur and were analyzed by flow cytometry for expression of GFP/human CD38; the other half of the organ and the second femur were analyzed by immunohistochemistry for expression of terminal desoxynucleotidyl transferase (Tdt; arrows indicate leukemia cells); rare, +, ++,+++indicate a rough quantification of the number of leukemic cells per field; **c** shows 1 representative image per week and post mortem analysis of bone marrow; in all mice, mid-abdominal signals are unspecific. **d** correlates results from imaging and FACs analysis in each mouse; correlation coefficient 0,86; Supplemental Figure 6 shows data on spleens.

**Figure 3:**
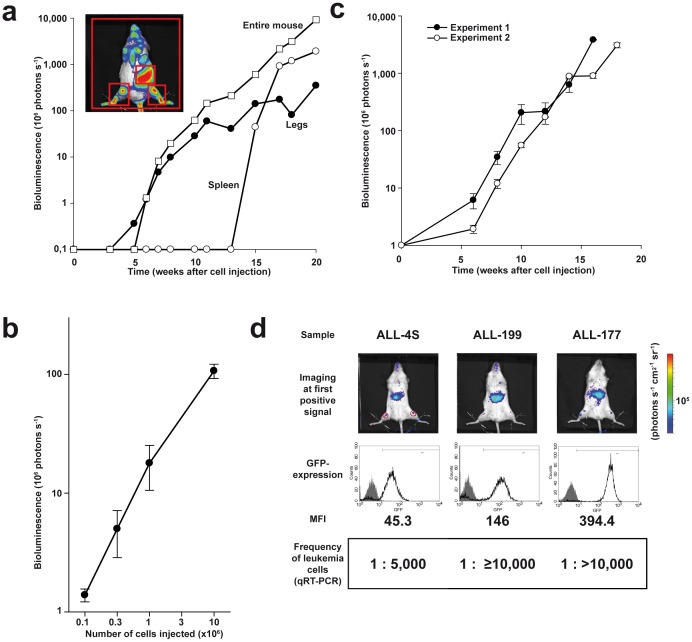
Quantification, quality parameters and visualization of minimal disease. a Procedure of quantification; images of the representative mouse shown in Figure 2a were quantified over time; 3 different regions were analyzed which are indicated with red squares in the image: (i) sum of 2 regions at the lower extremities (legs); (ii) part of the abdomen (spleen); (iii) the whole mouse body (entire mouse); **b** Logarithmic relation between cell number injected and light emission; all mice described in Figure 2b were imaged after 10 weeks and images were quantified; shown is the mean +/− standard error of the mean (SEM); **c** Imaging reveals logarithmic growth over time and low assay variances; 5×10^4^ ALL-177 cells per mouse were injected into groups of 5 mice each in two independent experiments performed 2 months apart; mice were imaged repetitively over time and images quantified; shown is the mean of each group +/− SEM; **d** Imaging enables monitoring minimal disease; ALL-4S, ALL-177 (5×10^4^ cells/mouse) or ALL-199 (1×10^4^ cells/mouse) with expression level of GFP as indicated (middle panel, mean fluorescence intensity - MFI) were injected into 7 mice per group; engraftment was considered positive at light emission above 6×10^4^ photons per second in the ROI indicated (see Methods for details) (upper panel shows one representative mouse); 4 of 7 mice were sacrificed and analyzed; shown are the frequencies of leukemia cells in bone marrow as determined by quantitative real time polymerase chain reaction (qRT-PCR) as mean per group of mice; see also Supplemental Figure 10. In all mice, mid-abdominal signals are unspecific.

Bioluminescence in vivo imaging using GLuc visualized engraftment of patient-derived ALL cells in mice first in the lower extremities where bones and bone marrow are located directly under the fur (Figure 2a). Over time, other bones such as sternum and jaw bones emitted light. Only at a late stage of disease, inner organs like spleen became visible. Leukemia-specific liver signals were 1 or 2 orders of magnitude more intensive compared to the unspecific, substrate-specific liver signal. Thus, GLuc-based in vivo imaging enabled the detailed monitoring of single animals over time.

Likewise, light emission strongly depended on the number of cells injected, indicating that both kinetic and dose response were clearly represented by in vivo imaging in this model (Figure 2b). The growth pattern of leukemia in mice was highly similar between the samples of different ALL patients and independent from the subtype of ALL or clinical parameters (Supplemental Figure 4, Supplemental Table 1). Expression of transgenes was restricted to human leukemia cells sparing mouse recipient cells as all GFP-expressing cells were positive for expression of human leukemia-specific antigens such as CD38 and CD45 (Supplemental Figure 5 and data not shown).

**Figure 4:**
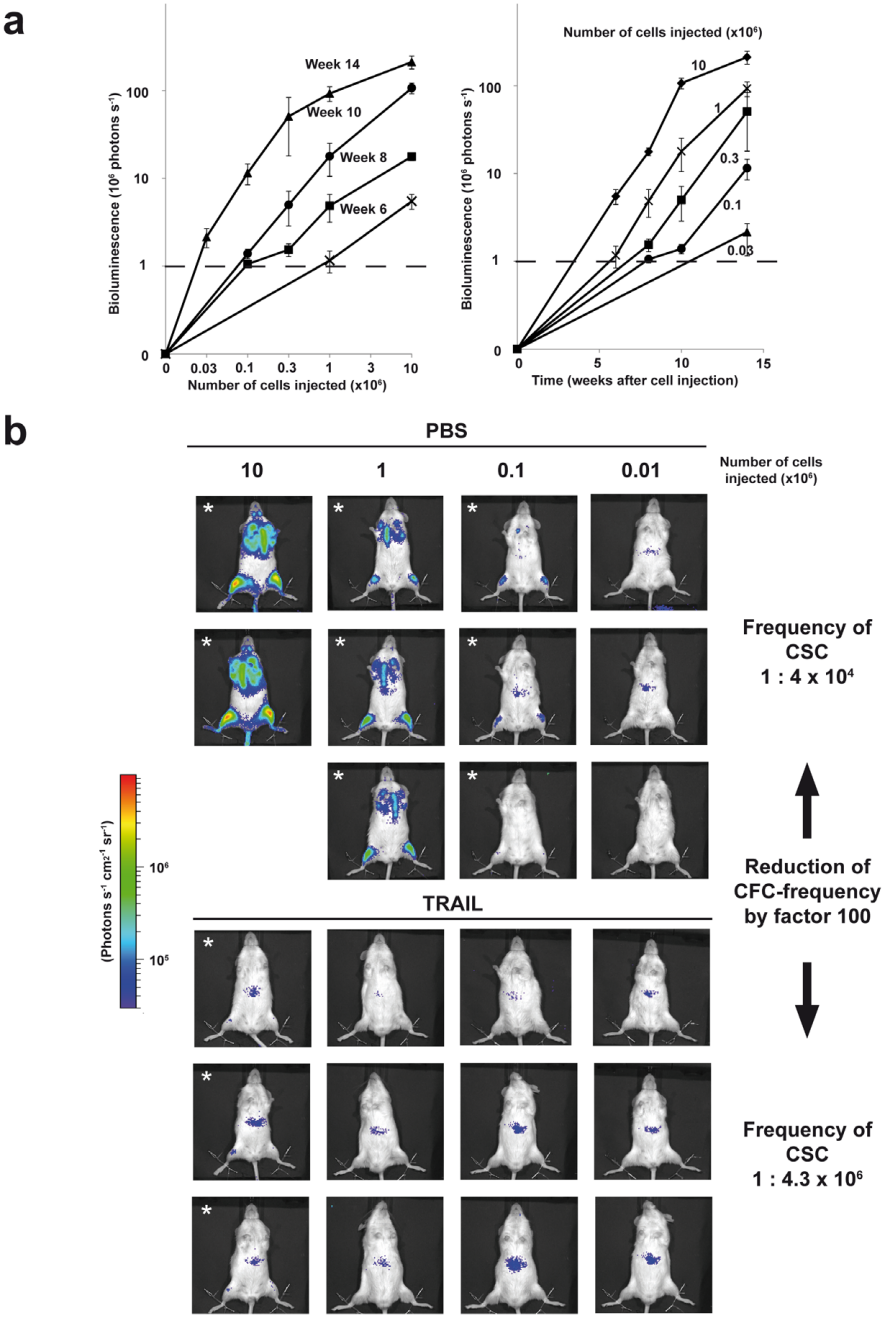
Imaging-based quantification of leukemia initiating cell frequencies. a Imaging visualizes dependence of leukemic growth on both time and cell numbers; experiment shown in Figure 2b was followed up over time in all groups injected with the different cell numbers; depicted is the quantification of imaging as mean of each group +/− SEM; **b** Imaging enables convenient determination of CSC frequencies; ALL-54 cells were freshly isolated from a mouse spleen, seeded at 10^6^ cells/ml and stimulated in vitro with PBS or TRAIL (1 µg/ml). After 48 hours, cells were serially diluted based on the cell concentration seeded at the beginning of the experiment and injected into groups of 2–3 mice. After 8 weeks, mice were imaged and analyzed for leukemic engraftment (defined using signals of legs only, identically as in Figure 3D; engraftment is indicated with a star); frequency of leukemia initiating cells was calculated out of engraftment rates using Poisson statistics. In all mice, mid-abdominal signals are unspecific.

**Figure 5:**
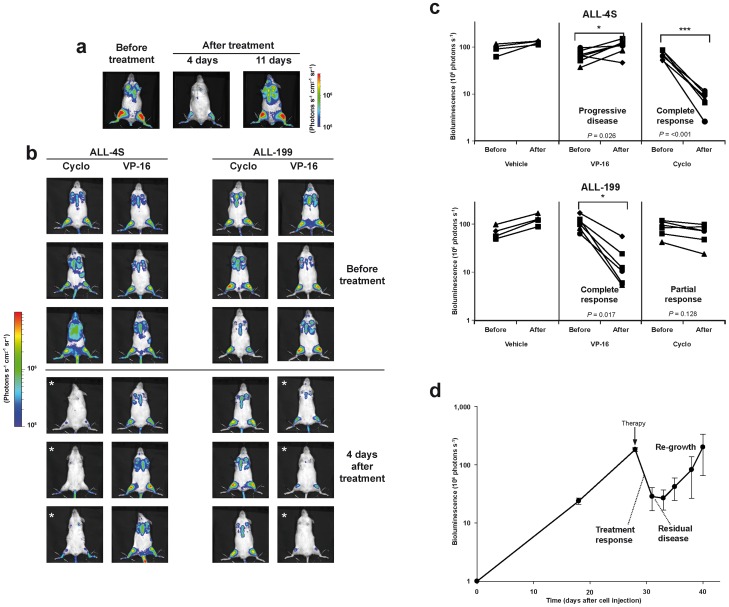
Precise quantification of individual treatment effects and monitoring of distinct clinical stages. a Imaging visualizes treatment-induced cell loss and regrowth; 10^5^ ALL-50 cells/mouse were injected into 10 mice which received a single intraperitoneal dose of Etoposid (VP-16; 50 mg/kg) in week 6 after tumor cell injection, except the control mouse which was treated with PBS. Animals were imaged before treatment (pre-treatment) and 4 and 11 days after treatment; shown is one representative mouse; all mice are shown in Supplemental Figure 11; **b, c**Imaging visualizes different sensitivities of individual samples towards treatment; ALL-199 (1×10^4^ cells/mouse) or ALL-4S (5×10^4^ cells/mouse) were injected into 16 mice; mice were randomized in week 4 into one control (n = 4) and two experimental groups (n = 6 each). Control mice received buffer injection, while the other groups were treated once intraperitoneally with either Etoposid (VP-16; 50 mg/kg) or cyclophosphamide (Cyclo; 150 mg/kg) as indicated. Mice were imaged directly before and 4 days after treatment; shown are 3 representative mice of each treatment group before and 4 days after treatment (**b**); shown is the result of quantification of the images; each line represents a single mouse (**c**); * *P*<0.05, *** *P*<0.001, Mann-Whitney Rank Sum Test; **d** Imaging visualizes disease stages known from patients; 15 mice were injected with 1×10^6^ ALL-199 cells/mouse and treated once intraperitoneally with Etoposid (VP-16; 50 mg/kg) in week 4; after treatment, mice were imaged three times per week; shown is the mean +/− SEM of image quantifications.

To reassure that imaging reliably visualized the leukemic disease, in vivo imaging data were correlated to conventional post mortem readouts. Single mice engrafted with sample ALL-50 were sacrificed weekly and bone marrow and spleen were analyzed by flow cytometry and immunohistochemistry to quantify leukemic cells. Imaging was more sensitive in detecting leukemic infiltration in bone marrow than in spleen arguing towards the highest sensitivity of imaging in visualizing ALL in bone marrow located directly under the fur (Figure 2c and Supplemental Figure 6; similar data not shown for sample ALL-4S, ALL-177 and ALL-199). Light emission correlated well to leukemic infiltration of bone marrow as measured post mortem by FACs analysis (correlation coefficient of 0,86; Figure 2d), but was unable to reliably detect leukemic infiltration in spleens (correlation coefficient of 0,65; Supplemental Figure 6). At late stages of leukemia disease, imaging revealed a diffuse leukemic infiltration throughout the entire mouse body which was paralleled by GLuc-based light emission from isolated organs such as bones, spleen, liver, peripheral blood, lungs and brain (Supplemental Figure 7). Taken together, imaging precisely visualized the clinical course of leukemia in single mice over time with good correlation to conventional post mortem readout parameters.

### Quantification, Logarithmic Growth and Quality Parameters

To quantify light emission, regions of interest were defined and analyzed using the Living Image software 4.0 (Caliper Life Sciences). In close correlation to the visual impression, quantification revealed first engraftment in a region covering the lower extremities, while inner organs became visible at late time points followed by extremely rapid signal increase (Figure 3a). For further studies, light emission of the entire animals was quantified as it represented the most reliable average value.

Quantification of light emission revealed a strictly logarithmic growth of patient-derived leukemia cells in mice covering up to 4 orders of magnitude (Figure 3a). Similarly, the relation between cell number injected and light emission was logarithmic (Figure 3b).

Individual leukemias were obtained from patients with very different clinical parameters, including primary disease and relapse, and contained completely different cytogenetic and molecular alterations (Supplemental Table 1). Light emission quantification of ALL samples showed that all samples grew in a logarithmic pattern in mice (Supplemental Figure 8 and data not shown). Thus, logarithmic growth appears as general principle how human leukemia cells behave in mice. Nevertheless and according to published data [19], the growth rate in mice differed markedly between the samples with sample ALL-177 showing a slower growth rate compared to most other samples (Supplemental Figure 8). Thus, bioluminescence in vivo imaging represents a useful tool to study inter-sample heterogeneity in growth kinetics.

To control quality parameters of the model, assay variances were estimated. Imaging showed a highly reproducible growth of leukemia in mice with surprisingly low intra- and inter-assay variances considering that an in vivo model was studied (Figure 3c, Supplemental Figure 9). Taken together, quantification of in vivo imaging revealed a strictly logarithmic growth of all individual leukemias in mice with high reliability and marginal standard errors.

### Visualization of Minimal Disease

Upon anti-leukemia treatment, most patients suffering from ALL accomplish complete morphological response. Nevertheless, minimal residual disease (MRD) represents a major clinical threat as MRD is difficult to treat and often followed by relapse. Appropriate preclinical models to study MRD are required [20].

To test, whether GLuc-based in vivo imaging could visualize MRD, its sensitivity was measured. Groups of mice were engrafted with equal cell numbers of the same sample and were imaged three times weekly. Upon crossing a clearly defined detection threshold (signal above 6×10^4^ photons per second using defined criteria, see Methods for details), most mice were sacrificed and bone marrow and peripheral blood analyzed by flow cytometry, quantitative real time PCR and immunohistochemistry. All remaining mice showed constant light increase similar to the kinetic shown in Figure 2a leading to overt leukemia (Supplemental Figure 10).

In all 3 samples tested, imaging was able to detect below 1 leukemia cell in 5,000 mouse bone marrow cells, while in one sample the detection threshold was even below 1 in 10,000 cells (Figure 3d and Supplemental Figure 10). Estimating the reported 10^9^ normal bone marrow cells per mouse, imaging allowed the visualization of less than 100.000 absolute leukemia cells per mouse. Detection sensitivity directly correlated with the expression level of the transgenes. At this early stage of first light emission, no leukemia cells were detected in peripheral blood using either flow cytometry or quantitative real time PCR. Thus, conventional readouts in peripheral blood did not allow follow up of leukemia in living mice at this stage. Instead, GLuc-based bioluminescence imaging revealed very high sensitivity for detecting human leukemic cells in mice which now allows studies, e.g., on MRD.

### Imaging-based Quantification of Leukemia Initiating Cell Frequencies

An important current concept in cancer research states that only cancer stem cells (CSC) are able to maintain tumor growth and therefore represent the most important targets for anti-cancer therapy [21]. The limiting dilution transplantation assay (LDTA) still represents the standard method for studying CSC [22]. LDTA functionally tests the CSC-defining ability of human tumor cells to induce tumors in mice. So far, performing LDTAs is hampered by the requirement of high mouse numbers.

Bioluminescence in vivo imaging allowed observing each group of mice within the LDTA repetitively over time (Figure 4a). Thereby, imaging increased the reliability of the measurements. Furthermore, it clearly visualized the dependence of CSC-frequency on engraftment time in mice.

Imaging-guided straightforward quantification of CSC-frequencies in LDTA enabled comparing untreated with treated patient-derived ALL cells using a rational number of mice. As an example, in vitro treatment of ALL-54 cells with TRAIL (TNF-related, apoptosis-inducing ligand) prior to transplantation significantly reduced engraftment of cells in mice (Figure 4b). These data reproduced our recently published finding that in vitro treatment with TRAIL reduces engraftment of leukemia cells in mice [23].

Taken together, imaging significantly facilitated performing LDTAs thus allowing the broader use of this assay in the future, e.g., to study drug sensitivities of leukemic CSC.

### Precise Quantification of Individual Treatment Effects

Treatment failure and relapse represent the most important challenges in anti-cancer treatment. Sophisticated preclinical models are required for testing novel therapeutic approaches addressing these threats in order to prepare their translation into the clinics.

Individual leukemias were grown until advanced disease (10^8^ photons per second per entire mouse). Imaging allowed starting treatment in mice with equal tumor burdens, although rarely mice had to be excluded, as leukemia grew highly homogenously in mice (compare Supplemental Figure 9). Mice were treated with conventional cytotoxic drugs by systemic bolus injections in concentrations modeling drug doses typically applied in patients [24]. Few days after treatment with an effective drug, light emission from mice was significantly reduced (Figure 5a). Thereby, low variances were found within a group of equally treated mice enabling highly reliable quantification of the therapeutic effects (Supplemental Figure 11a and 11c). As light emission persisted after therapy, the single drug application had reduced, but not eliminated leukemia. In our trials, a single bolus injection reduced light emission at maximum by 1 order of magnitude suggesting that at least 10% of tumor cell persist after a single treatment (data not shown) [25]. The effectiveness of drugs was independent from the leukemic burden of the mice within the log growth phase. Therapeutic reduction of leukemia was followed by tumor re-growth shortly afterwards (Figure 5a).

Imaging results correlated well with data obtained by conventional post-mortem readout parameters in most organs (Supplemental Figure 11b and 11d). Treatment nearly eliminated tumor cells from peripheral blood, while imaging visualized the remaining tumor burden post treatment (Supplemental Figure 11e). Due to limited sensitivity, conventional measurement in peripheral blood appears overestimating therapeutic effects, although this readout was most frequently used for disease monitoring until today.

In our model, each mouse harbors the leukemia of an individual patient. Imaging-guided preclinical treatment trials in mice revealed that individual ALL samples retained individual sensitivities towards conventional cytotoxic drugs. While the T-ALL sample ALL-4S was sensitive towards treatment with Cyclophosphamide, it was resistant towards Etoposid; in contrast, the B-ALL sample ALL-199 was resistant towards Cyclophosphamide, but sensitive towards Etoposid (Figure 5b). Accordingly, treatment with Cyclophosphamide prolonged survival of ALL-4S, but not of ALL-199 bearing mice, while Etoposid prolonged survival of ALL-199, but not of ALL-4S bearing mice (data not shown). Thus, in vivo imaging was able to visualize specific drug sensitivities of each individual sample towards a given anti-cancer treatment shortly after treatment within the preclinical trial.

Most importantly, imaging was able to quantify therapeutic responses and to visualize tumor regrowth. Due to low assay variances, imaging allowed distinguishing treatment failure/drug resistance/progressive disease from partial or complete response (Figure 5c). As additional advantage, imaging enabled quantifying the therapeutic effect already few days after treatment, while conventional readouts like survival require long incubation periods.

Taken together, in vivo imaging allowed the rapid, precise and individual quantification of treatment responses.

### Monitoring of Distinct Clinical Stages Upon Treatment in Mice

The sensitivity of tumor cells towards treatment depends on the disease stage at which the drug is given [26]. Therefore, preclinical models are required modelling distinct disease stages including MRD. Furthermore, time to tumor re-growth/time to tumor progression/time of progression free survival represents a prognostic parameter for patients, especially in MRD.

After treatment, imaging reliably quantified residual disease. Short-interval imaging revealed short “lag phase” of no-growth after a single application for certain, but not all drugs and samples (Figure 5d). In the later course of the disease, imaging revealed reappearance of a second logarithmic tumor growth phase modeling tumor regrowth. Thus, in vivo GLuc-based imaging allowed the precise preclinical modeling in mice of the complex clinical course and disease stages known from cancer patients.

Taken together, we have introduced and validated in detail GLuc-based bioluminescence in vivo imaging in the xenograft mouse model of individual patient’s ALL. Imaging was easy to perform and gave rise to highly sensitive and reliable results which enable the non-invasive accurate and detailed monitoring of disease progression and treatment responses.

## Discussion

GLuc-based bioluminescence in vivo imaging was introduced and intensively validated as novel readout parameter for the preclinical model of patient-derived ALL growing in mice. In vivo imaging allowed performing preclinical trials on a novel level of accuracy and precision including stage-specific therapy and quantification of treatment responses. In the future, improvement of the individualized ALL mouse model by in vivo imaging will allow performing preclinical trials more exactly and in more detail.

Imaging allowed modeling disease stages in mice which represent current challenges in the clinics such as minimal residual disease and tumor regrowth. Imaging was highly sensitive and continuous and correlated well with post mortem results regarding tumor distribution. Assay variances were minimal which will reduce the number of animals required per experiment. In addition, tumor growth was orthotopic and homogenous and tumor cells were derived from individual patients with genetically defined tumors; limiting dilution assays were easily visualized to study drug sensitivity of leukemia initiating cells. Taken together, GLuc-based imaging will allow performing high quality and convenient preclinical treatment trials in the individual mouse model of ALL in the future.

The most important challenges in cancer treatment represent treatment failure and relapse. Using in vivo imaging, treatment failure is easily detectable in the first days after treatment as light emission continues increasing despite of therapy; short-period treatment quantification will allow speeding up preclinical treatment trials. Tumor regrowth is visualized by quantification of post-therapeutic residual disease followed by monitoring increase in light emission. Thus, our model is able to exactly map both important clinical challenges for preclinical trials.

In addition to bioluminescence imaging, fluorescence imaging represents an interesting alternative with a better anatomical resolution, especially using near- infrared fluorochromes [12], [27]. Even though leukemic cells used in our studies also expressed GFP, GFP proved non-suitable for in vivo imaging due to the known high tissue autofluorescence signals emitted at the same wave length [28]. Fluorescent probes with near-infrared and far-red light emission have been recently developed which might be more suitable for small animal imaging [29]. In our hands, leukemia cells did not express sufficient levels of the mRaspberry protein (data not shown), which might have been due to reported cellular toxicity induced by the fluorochrome [30].

Recently, two groups published the use of firefly luciferase for in vivo imaging of transplanted individual ALLs [31], [32]. While one publication used the method without commenting on methodological details [31], the second study examined growth kinetics and showed an imaging-guided treatment trial starting at mainly invisible leukemia load [30]. The novelty of the present study lies in (i) the use of GLuc as lighter luciferase; (ii) the detailed methodological validation of the technique including its sensitivity allowing in vivo studies on MRD in the future; (iii) the prove that treatment trials can now be performed at visible leukemic burden (iv) the ability of imaging to diagnose treatment resistance within days after treatment allowing secondary interventions; (iv) the prove that routine clinical outcome parameter can now be monitored in mice.

The major advantage of using GLuc instead of firefly luciferase for in vivo imaging is the markedly increased light emission from superficial organs such as bone marrow in the lower extremities of mice [18]. Hence, GLuc-based imaging proved superior to firefly luciferase-based imaging in the context of T-cell imaging [13]. Therefore, we argued that GLuc might be more sensitive compared to firefly luciferase for imaging of patient-derived ALL and therefore used GLuc in our experiments.

Leukemic disease serves as a suitable model disease for cancer in general since leukemia cells are easier in handling compared to solid tumor cells. Many important research discoveries in cancer biology were first described in leukemia; for example, oncogenic mutations are best characterized in acute myeloid leukemia [33] and; the cancer stem cell concept evolved upon research on leukemias [34]. Transferring its role from studying tumor biology to anti-tumor therapy, leukemia might now play a pivotal model disease for directing anti-cancer treatment towards individualized and disease stage-specific strategies.

Taken together, GLuc-based in vivo imaging in the individualized preclinical model of ALL enables performing treatment trials on a novel level of accuracy and precision. It enables quantifying therapy effects and remaining disease burdens as well as exact modeling of distinct disease stages. The model facilitates the detailed preclinical analysis of novel therapies for preparing their translation into the clinics. The model allows preclinical trials addressing the most demanding current clinical challenges, such as treatment failure, resistance in leukemia initiating cells, minimal residual disease and relapse.

## Supporting Information

Supporting Information S1 (PDF)
