# Supplementary material for: In Vivo Imaging Enables High Resolution Preclinical Trials on Patients’ Leukemia Cells Growing in Mice
Source: PLoS One. 2012 Dec 31;7(12):e52798. doi: 10.1371/journal.pone.0052798 (PMC3534096; doi:10.1371/journal.pone.0052798)
Supplement: Supporting Information S1 — (PDF) [file pone.0052798.s001.pdf]

Supplemental Information

In vivo imaging enables high resolution preclinical trials on patients' leukemia cells growing in mice

by Nadia Terziyska, Catarina Castro Alves, Volker Groiss, et al.

2	pages	Supplemental Materials and Methods
1	page	Supplemental Results
1	page	References for the Supplemental Information
11		Supplemental Figures
2		Supplemental Tables

Correspondence: Irmela Jeremias, MD
Department of Gene Vectors
Helmholtz Zentrum München
German Research Center for Environmental Health
Marchioninistraße 25
81377 Munich
Germany
irmela.jeremias@helmholtz-muenchen.de
tel. +49-89-7099-424
fax +49-89-7099-225

Supplemental Methods

The NSG mouse model of individual ALL

Primary ALL blasts were obtained from children treated at the Ludwig Maximilians University Children's Hospital. NSG mice (NOD / scid, IL2 receptor gamma chain knockout mice) were obtained from The Jackson Laboratory (Lund, Sweden). The animal model was performed as described(1). Briefly, fresh primary childhood ALL cells were isolated by Ficoll gradient centrifugation from peripheral blood or bone marrow aspirates that had been obtained from leftovers of clinical routine sampling before onset of therapy. 1-10 million ALL cells were injected into 6-8 weeks old NSG mice via the tail vain. Engraftment was monitored by weekly flow cytometry measurement of human cells in peripheral blood; time to engraftment was defined as time period until 1 % of blood cells were leukemic. Engrafted human ALL cells were isolated from spleens of diseased mice by Ficoll gradient centrifugation and injected into next generation of mice or subjected to in vitro experiments.

Flow cytometry and histology

The analysis of cell apoptosis, surface markers and GFP expression was performed with FACSCalibur cytometer using CellQuest Pro software (BD Biosciences, Franklin Lakes, NJ, USA). The following antibodies were used: CD22 IgG1 PE, CD19 PE-Cy5.5 IgG1, CD10 APC IgG1, CD7 APC IgG2a, CD5 PE-Cy5.5 IgG1, HLA-DR (Class II) PE-Cy5.5 IgG2b (Life Technologies, Darmstadt, Germany); CD4 PE IgG1 (Dako, Hamburg, Germany) and CD19 FITC IgG1, CD20 FITC IgG1, CD34 PE IgG1, CD38 PE IgG1 (Becton Dickinson, Heidelberg, Germany).

Immunohistochemistry analysis was performed as described previously(2), using anti-human terminal desoxynucleotidyl transferase (Tdt; Dako, Hamburg, Germany) and anti-human CD10 antibodies (Novocastra, New Castle, UK).

In vitro stimulation

To exclude a potential influence of lentiviral transduction on drug sensitivity, four samples (ALL-199, ALL-4S, ALL-50 and ALL-177) were tested comparing untransduced and transduced cells. Patient-derived leukemia cells were freshly isolated from mouse spleens, purified and cultured as described(3). Several cytotoxic

drugs (Vincristine, Etoposid, Amsacrine, Cytarabine, Dexamethasone, Doxorubicine, Epirubicine, Methotrexate, Cyclophosphamide and 6-Thioguanin) were tested in vitro in clinically relevant concentrations; as a control, cells were incubated with solvents alone. After 72 hours, cell apoptosis was measured by flow cytometry using both forward side scatter analysis and propidium iodide staining yielding similar results(4).

Quantitative real time polymerase chain reaction (q RT-PCR)

Real-time quantitative PCR was performed and interpreted according to the guidelines developed within the European Study Group for MRD detection in ALL (ESG-MRD ALL)(5). In detail, total DNA was isolated from 5 million bone marrow cells and from 150-200 µl whole blood using the High Pure PCR Template Preparation Kit (Roche, Mannheim, Germany). TaqMan probes were used on a LC480 machine (Roche, Mannheim, Germany). For the standard curve, DNA was prepared from bone marrow of a mouse with > 99% GFP positive human leukemia cells and diluted in tenfold serial dilutions. Quantification was performed using this standard curve and triplicate follow-up samples including 250 ng DNA in each reaction. As target for detection of human leukemic cells in mice samples, we used a beta-globin Taq-Man based RQ-PCR assay that specifically detects human DNA with the following primers and probes: Primer forward 5‘-CTG ACA CAA CTG TGT TCA CTA GC; primer reverse 5’- TAT TGG TCT CCT TAA ACC TGT CTT G and TM-probe Fam-5’- CCC ACA GGG CAG TAA CGG CAG AC-3’-BHQ-1.

Limiting dilution transplantation assay

ALL-199 cells were freshly isolated from a mouse spleen, seeded at 10^6cells/ml and stimulated in vitro with PBS or vincristine (VCR) (300 nM). After 48 hours, cells were serially diluted based on the cell concentration seeded at the beginning of the experiment and injected into 4 groups of 2-3 mice. After 4 weeks, mice were imaged and analyzed for leukemic engraftment (defined as described above and indicated with a star) without post mortem analysis of animals; frequency of cancer stem cells (CSC) was calculated out of engraftment rates using Poisson statistics.

Supplemental Results

Generation of GLuc -expressing patient-derived ALL cells

Gaussia luciferase (GLuc) was chosen due to its high light emission in comparison to other luciferases. GLuc was used in its membrane-bound form fused to the transmembrane part of human CD8 in order to prevent secretion(6). As patient-derived ALL cells are difficult to transfect, the GLuc construct was cloned into the lentiviral pCDH vector harboring an expression cassette for GFP for ex vivo cell monitoring and sorting. For favorable expression in hematopoietic cells, the EF1alpha promoter was used (Fig. 1a).

Primary tumor cells of 8 children with pre-B ALL and 1 child with T-ALL (for clinical data see suppl. Table 1) were engrafted via tail vein injection and passaged in non-obese diabetes / severe combined immune deficient mice lacking a functional IL-2 receptor common gamma chain (NSG mice). For lentiviral transduction, cells were freshly isolated from the spleens of mice and transduced overnight. After intensive removal of remaining viruses, cells were re-transplanted into mice for amplification and re-isolated from the spleens. All 9 patient-derived ALL samples were transduced by this approach, although transductin efficiency and level of transgene expression varied markedly between the samples (0,8% to 62% transduction efficiency, Fig. 1b and 1c).

To enrich transduced cells, samples were sorted using fluorescence activated cell sorting. All samples were enriched by flow cytometry once, except ALL-4S and ALL-50 which were enriched twice; after sorting, all samples had a transgene expression of above 90 % (data not shown).

Transgene expression remained stable over passaging arguing towards a successful transduction of leukemia stem cells and a non-toxic transgene expression (suppl. Table 2). Expression of GLuc and GFP did not alter important functional characteristics of patient-derived ALL cells. E.g., growth kinetics in mice, in vitro apoptosis sensitivity towards cytotoxic drugs or expression of cell surface markers varied between transduced and non transduced samples in the same range as it varied between unmodified passages in mice according to the known minor clonal evolution in this mouse model(7) (Fig. 1d, suppl. Fig. 1).

Taken together, lentiviral transduction enabled generating transgenic patient-derived ALL cells expressing marker proteins without altering their basic biological characteristics.

Supplemental References

(1) Liem NL, Papa RA, Milross CG, Schmid MA, Tajbakhsh M, Choi S, et al. Characterization of childhood acute lymphoblastic leukemia xenograft models for the preclinical evaluation of new therapies. Blood 2004;103:3905-3914.

(2) Kunder S, Calzada-Wack J, Holzlwimmer G, Muller J, Kloss C, Howat W, et al. A comprehensive antibody panel for immunohistochemical analysis of formalin-fixed, paraffin-embedded hematopoietic neoplasms of mice: analysis of mouse specific and human antibodies cross-reactive with murine tissue. Toxicol Pathol 2007;35:366-375.

(3) Ehrhardt H, Hofig I, Wachter F, Obexer P, Fulda S, Terziyska N, et al. NOXA as critical mediator for drug combinations in polychemotherapy. Cell Death Dis. 2012;3:e327.

(4) Ehrhardt H, Hacker S, Wittmann S, Maurer M, Borkhardt A, Toloczko A, et al. Cytotoxic drug-induced, p53-mediated upregulation of caspase-8 in tumor cells. Oncogene 2008;27:783-793.

(5) van dV, V, Cazzaniga G, Schrauder A, Hancock J, Bader P, Panzer-Grumayer ER, et al. Analysis of minimal residual disease by Ig/TCR gene rearrangements: guidelines for interpretation of real-time quantitative PCR data. Leukemia 2007;21:604-611.

(6) Santos EB, Yeh R, Lee J, Nikhamin Y, Punzalan B, Punzalan B, et al. Sensitive in vivo imaging of T cells using a membrane-bound Gaussia princeps luciferase. Nat Med 2009;15:338-344.

(7) Schmitz M, Breithaupt P, Scheidegger N, Cario G, Bonapace L, Meissner B, et al. Xenografts of highly resistant leukemia recapitulate the clonal composition of the leukemogenic compartment. Blood 2011;118:1854-1864.

a

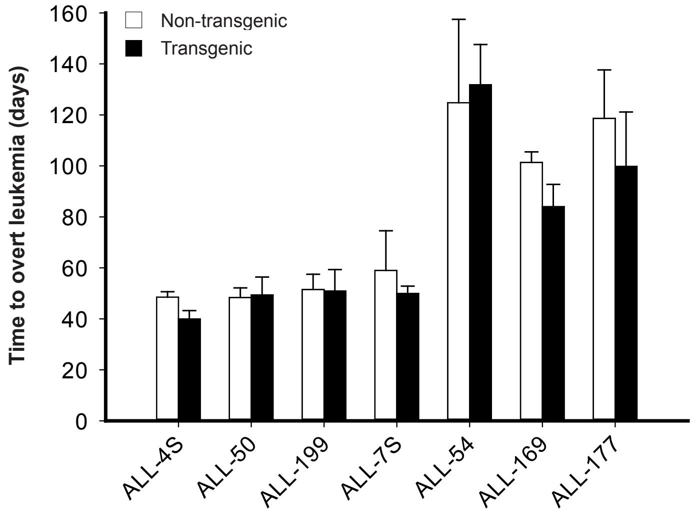

Suppl. Figure 1 Quality control of transgenic samples

Each sample was tested comparing the passage before lentiviral transduction with the passage used for imaging experiments after transduction and sorting. (**a**) Time period required to induce overt leukemia in mice (10^{10} photons per second per entire mouse), when similar tumor cell numbers were injected per sample; shown is the mean of an average of 3 (minimum 2, maximum 6) independent mice per sample + SEM.

b

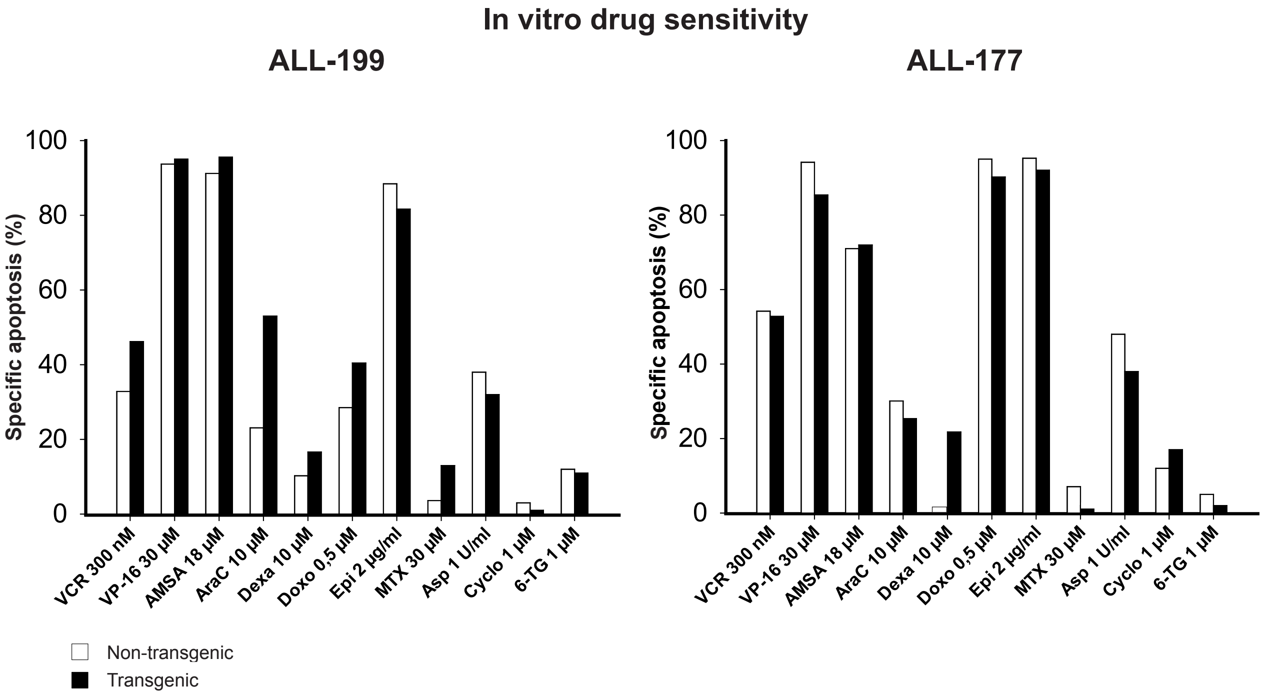

(**b**) Leukemic cells were freshly isolated from the spleens of diseased mice and stimulated with PBS or drugs indicated; after 48 hours, apoptosis was measured by flow cytometry and specific apoptosis was calculated as [(percentage of dead cells after drug treatment - percentage of dead cells after PBS treatment)] divided by (100 - percentage of dead cells after PBS treatment) times 100. The following drugs were used: VCR = Vincristine, VP-16 = Etoposid, AMSA = Amsacrin, AraC = Cytarabin, Dexa = Dexamethasone, Doxo = Doxorubicine, Epi = Epirubicine, MTX = Methotrexate, Asp = Asparaginase, Cyclo = Cyclophiosphamide, 6-TG = 6-Thioguanin; shown are 2 samples each tested in a single experiment as mean of duplicates.

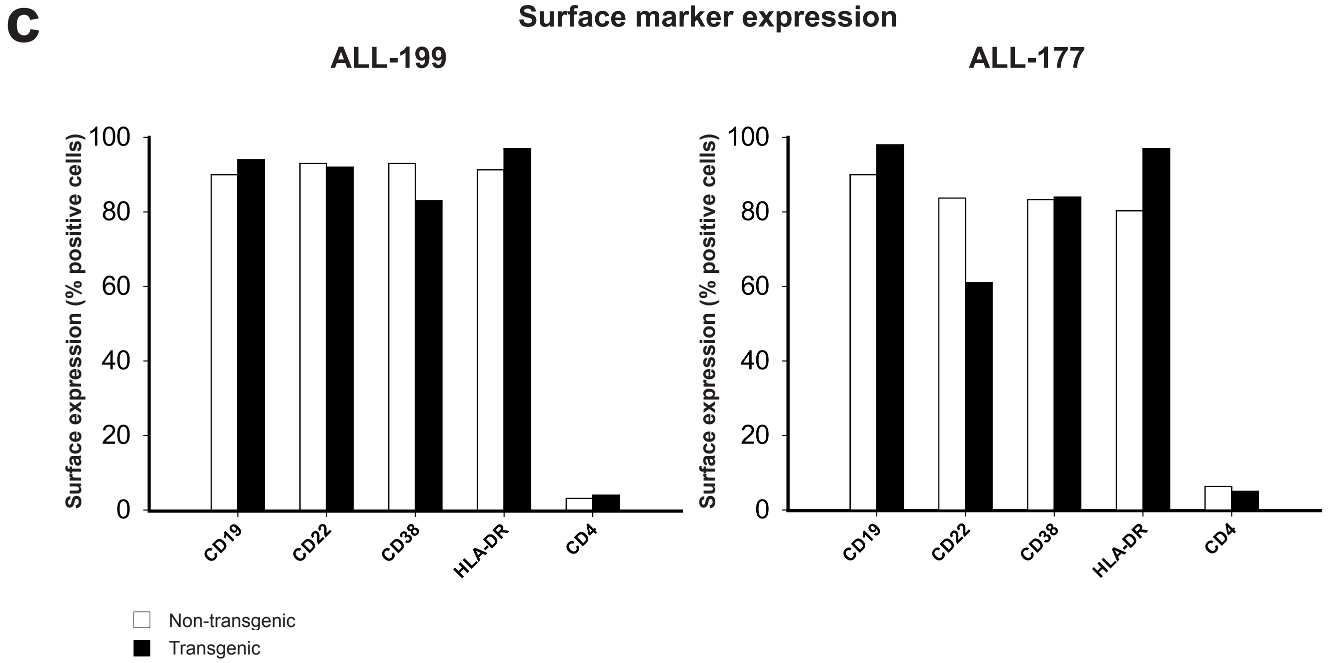

(**c**) Frozen cells were thawed and stained for the surface antigens indicated and analyzed by flow cytometry; shown is the percentage of cells expressing the defined antigens.

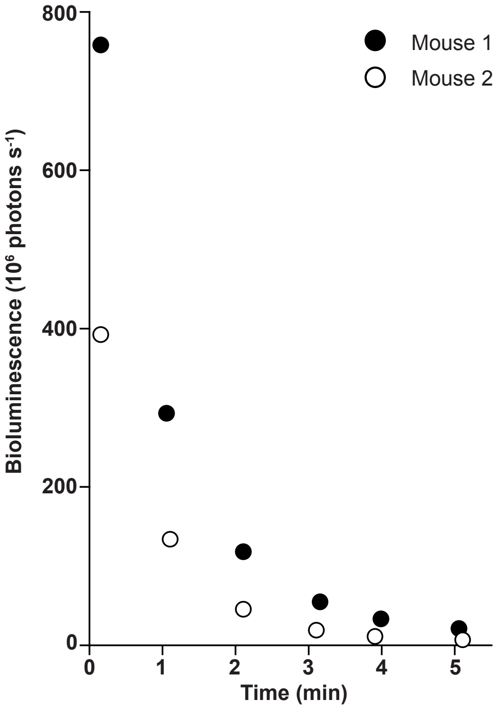

Suppl. Figure 2

Short light emission by GLuc

2 mice with advanced stage leukemia were injected with Coelenterazine and imaged repeatedly every minute for 15 seconds; images were quantified.

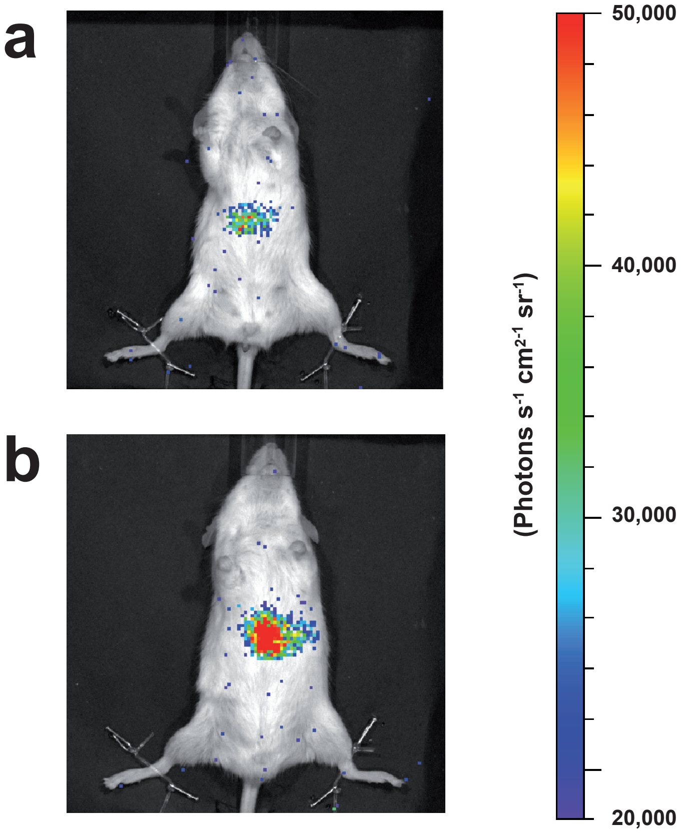

Suppl. Figure 3
Liver-related background signal induced by Coelenterazine
An untreated control mouse in the absence of leukemic cells (a) and a mouse injected with ALL-199 leukemia cells expressing GFP only in the absence of expression of GLuc (b) were imaged using Coelenterazine.

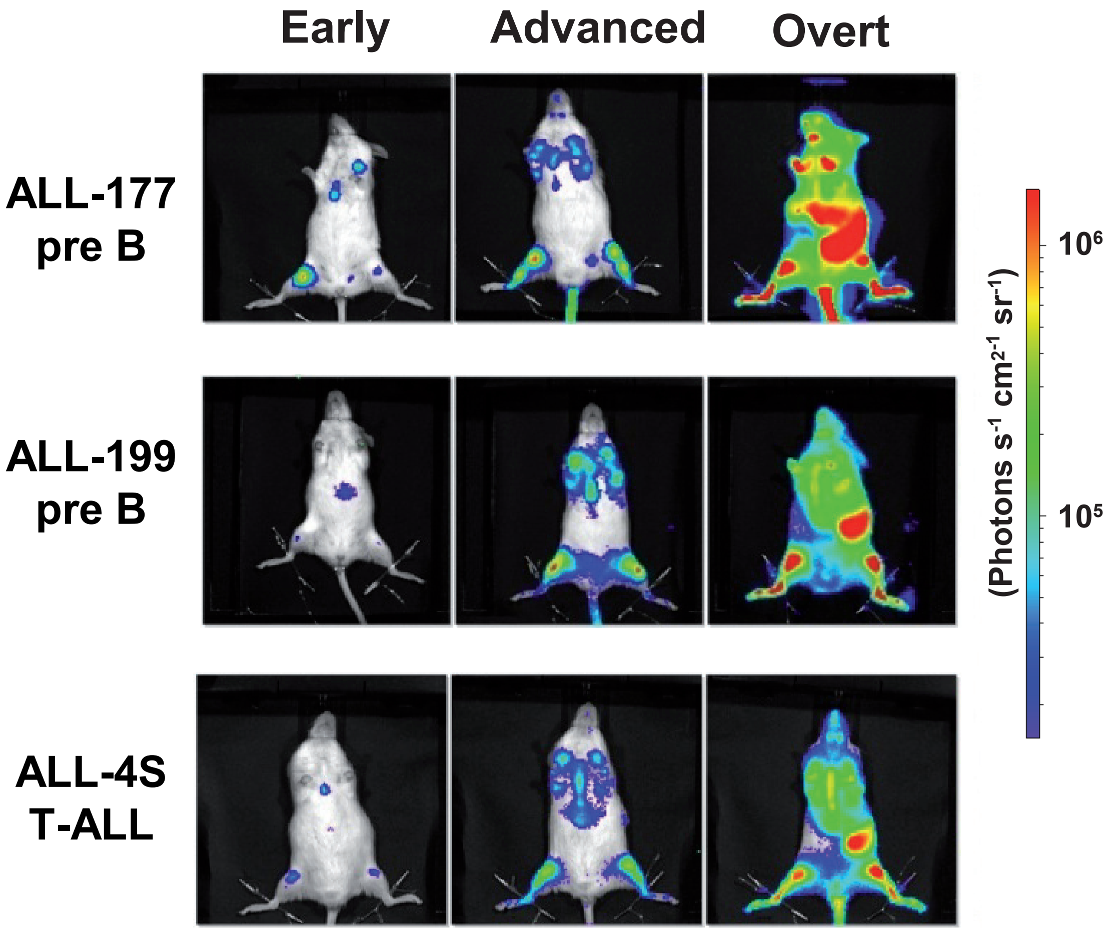

Suppl. Figure 4

Similar visual growth pattern of different ALL subtypes - pictures
3 ALL samples (cells per mouse: 10^4 in ALL-199, $5x10^4$ in both ALL-4S and ALL-177) were injected into groups of 5-10 mice and leukemic growth was followed by imaging; representative images are shown for the different stages of the leukemic disease.

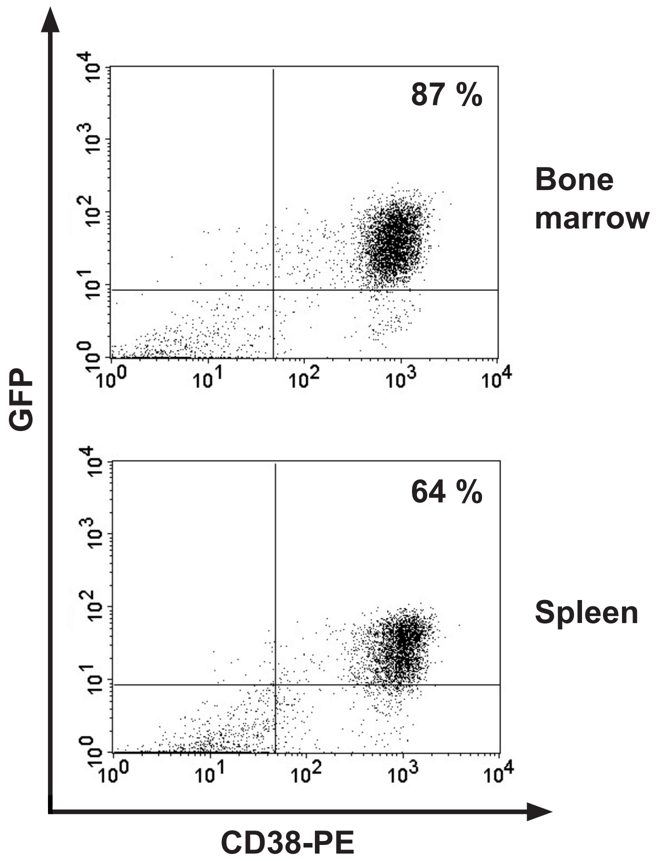

Suppl. Figure 5

Expression of transgene is restricted to human leukemia cells Isolated cells from organs of a mouse engrafted with 10^5 ALL-50 cells were stained with anti human CD38 antibody and measured by flow cytometry together with expression of transgenic GFP.

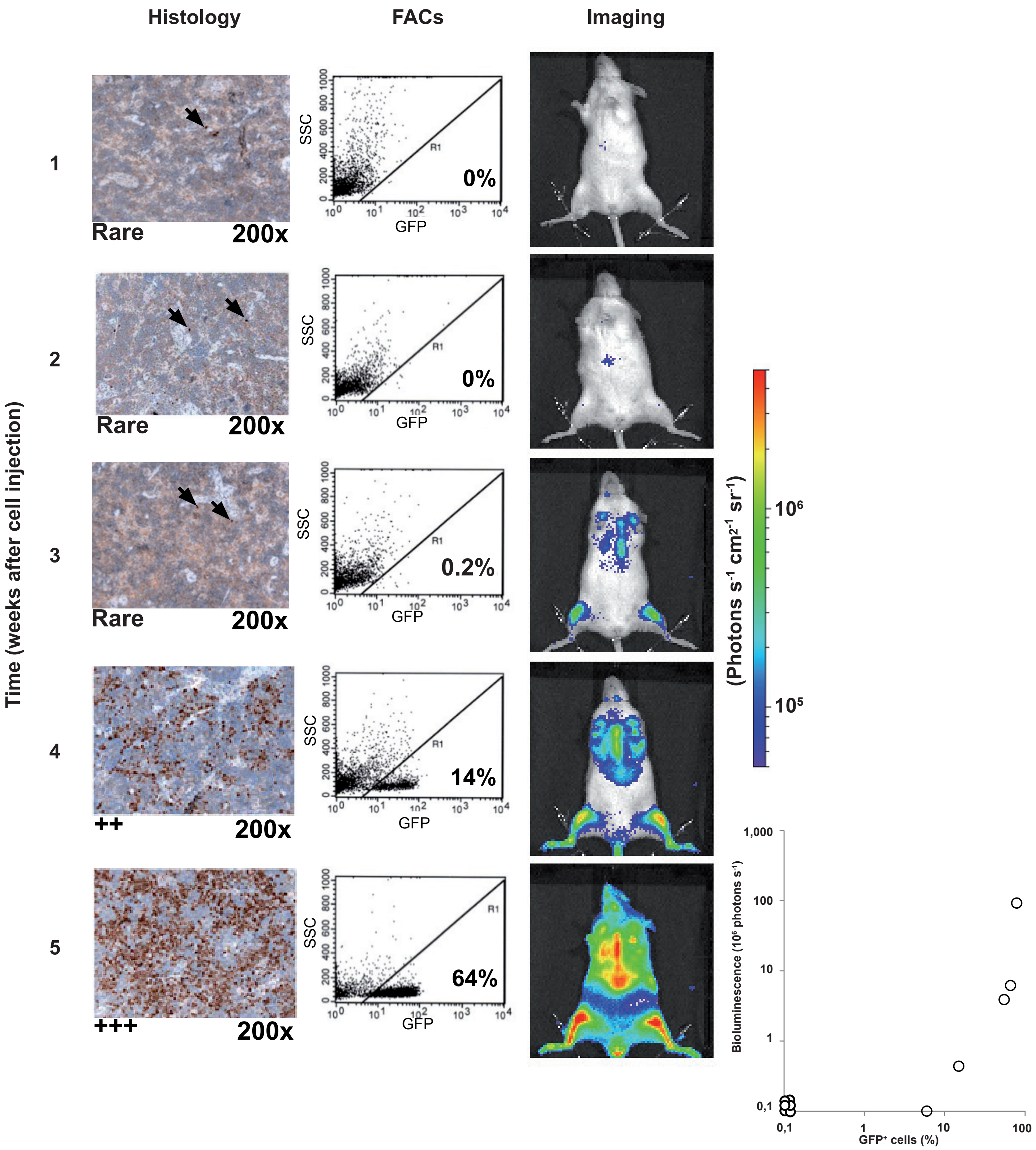

Suppl. Figure 6

Good correlation between imaging and post mortem readouts

12 mice were injected with 10^5 ALL-50 cells / mouse; each week 2 mice were imaged, sacrificed and organs were analyzed by flow cytometry for expression of GFP and by immunohistochemistry for expression of terminal desoxynucleotidyl transferase (Tdt) in bone marrow and spleen; rare, +, ++, +++ indicate a rough quantification of the number of leukemic cells per field; shown are data splenic cells; data on bone marrow are shown in Fig. 2c. Lower panel: Correlation of results from imaging and FACs analysis in each mouse; correlation coefficient 0,65.

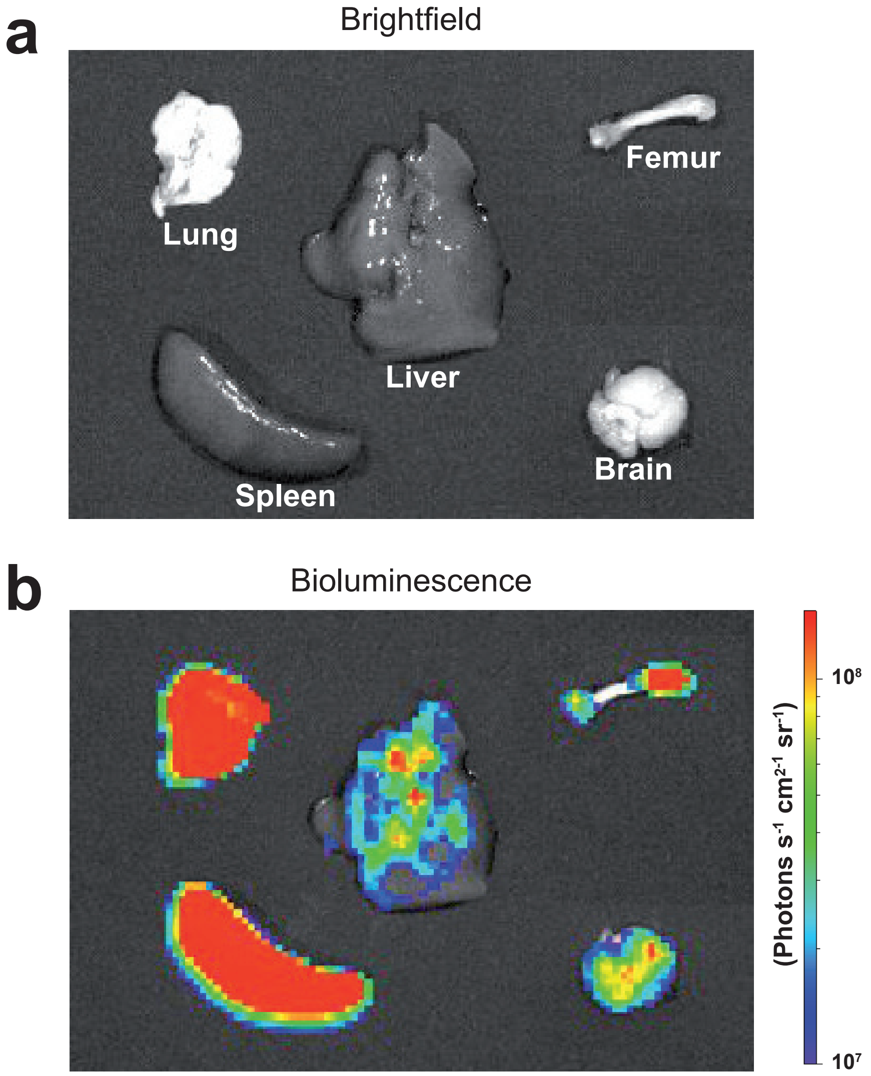

Suppl. Figure 7

Leukemic infiltration of numerous organs at late disease stages

Of the experiment shown in Figure 2a, time point 21 weeks after tumor cell injection, organs were isolated rapidly after in vivo imaging for ex vivo imaging at bright-field **(a)** and for bioluminescence imaging **(b)**; organs of a representative mouse is shown.

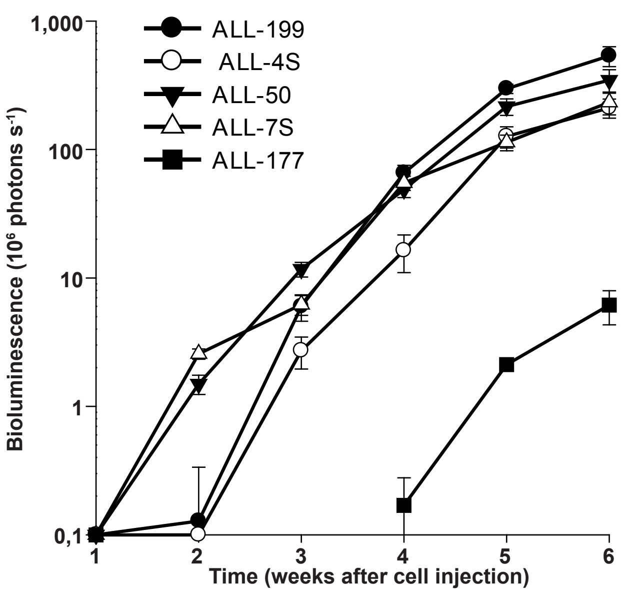

Suppl. Figure 8

Similar growth pattern of diffrent ALL subtypes - quantification

5 ALL samples (cells per mouse: 10^4 in ALL-199, 10^5 in ALL-50, $5x10^4$ in both ALL-4S and ALL-177, 10^5 in ALL-7S) were injected into groups of 5-10 mice and leukemic growth was followed over time by imaging; shown is the quantification of images as mean of each group +/- SEM.

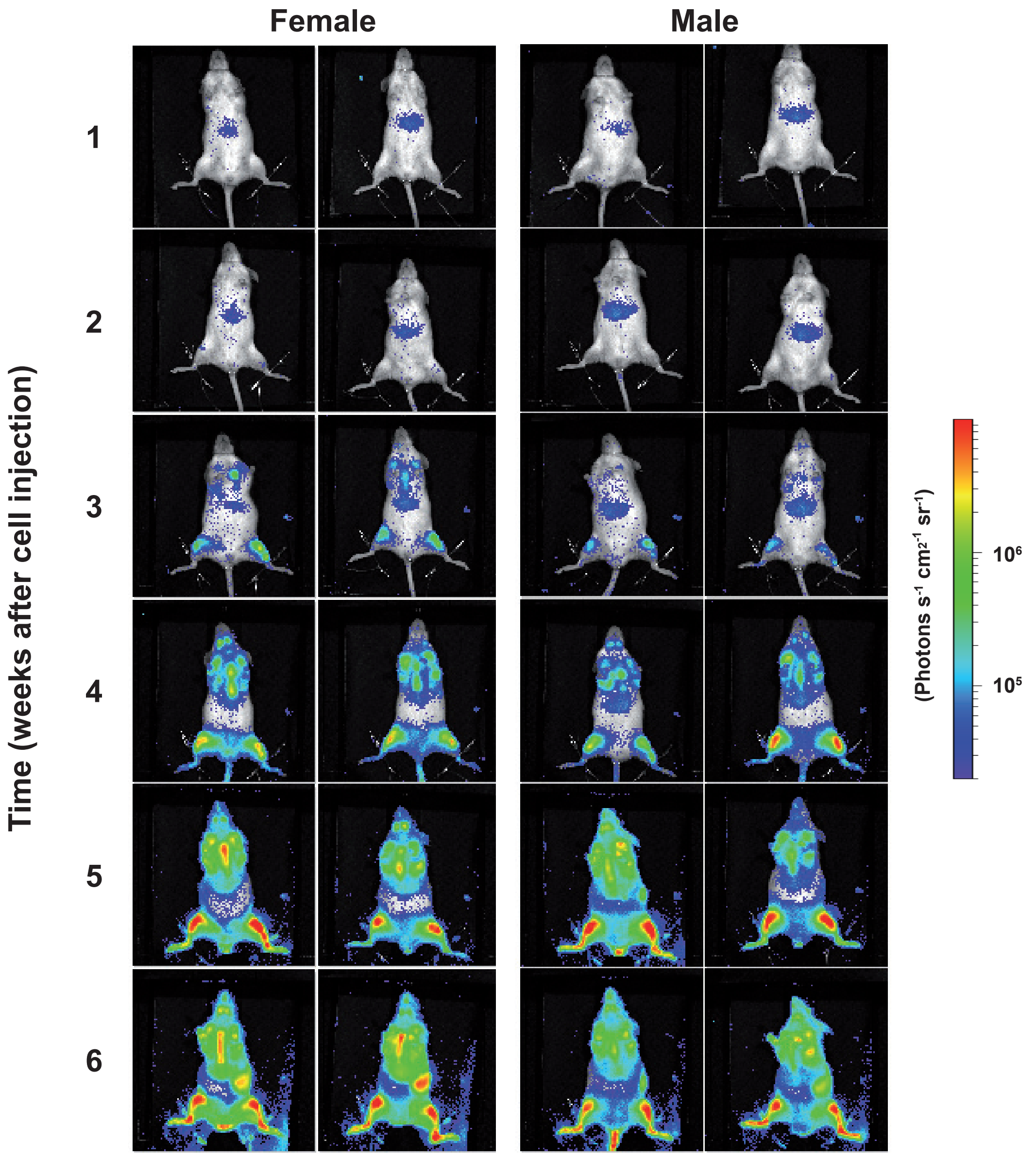

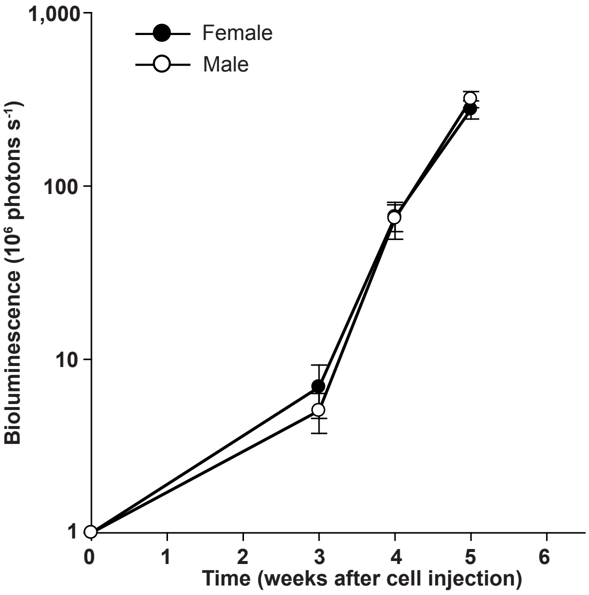

Suppl. Figure 9

Low intra-assay variances of leukemic growth in mice as quantified using imaging

2 female (left) and 2 male (right) mice were injected with 10^4 ALL-199 cells / mouse and imaged weekly; shown are the images (upper panel) and their quantification (lower panel; mean of both mice +/- SEM).

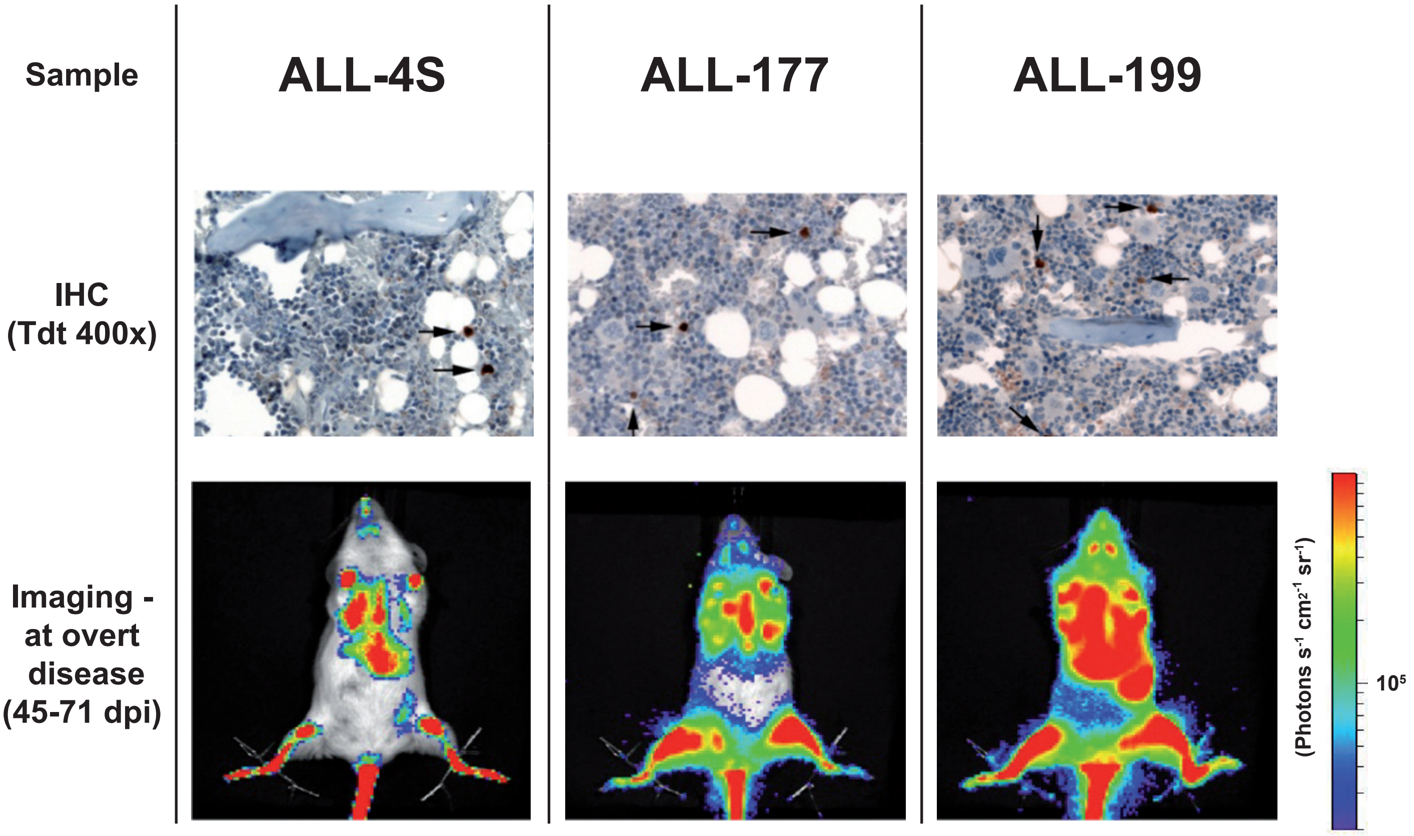

Suppl. Figure 10

Visualization of minimal disease

Supplemental data to the experiment described in Fig. 3d; ALL-4S, ALL-177 ($1x10^4$ cells / mouse) or ALL-199 ($1x10^4$ cells / mouse) were injected into 7 mice per group and imaged three times per week, when leukemic engraftment was expected; engraftment of leukemia was considered positive at light emission above 6 x 10^4 photons per second using defined criteria (see Methods for details) and was achieved 9-21 days after injection of cells; upon engraftment of leukemia, 4 of 7 mice of the group were sacrificed and analyzed; shown is immunohistochemistry of bone marrow of a representative mouse sacrificed directly after detection of first imaging signals (upper panel) and leukemic distribution at late disease stage in the control mouse (lower panel); Fig. 3d shows imaging at first visible engraftment, GFP expression and results of qRT-PCR.

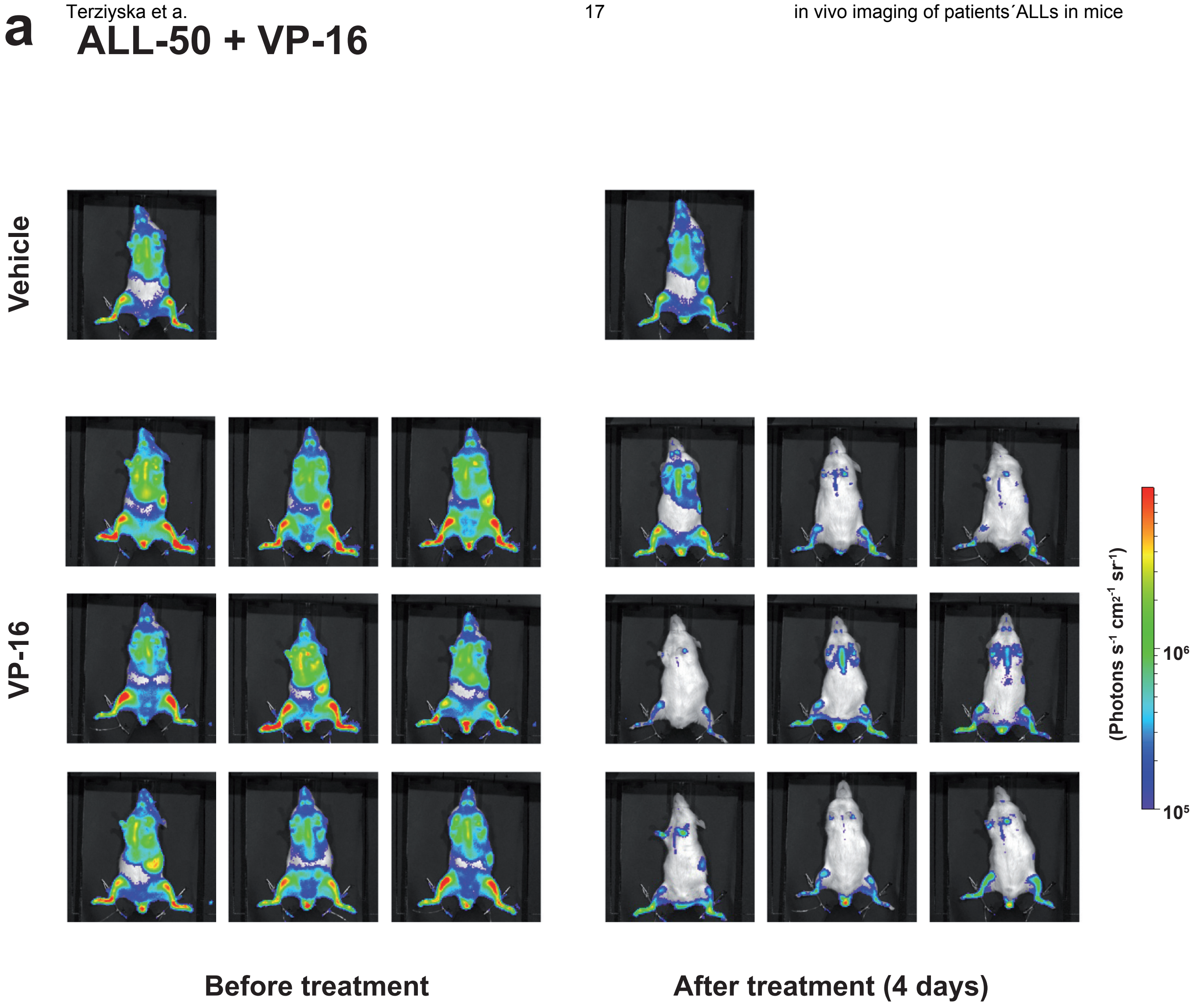
a
ALL-50 + VP-16
Vehicle
VP-16
Before treatment
After treatment (4 days)
(Photons s-1 cm2-1 sr-1)
10⁶
10⁵

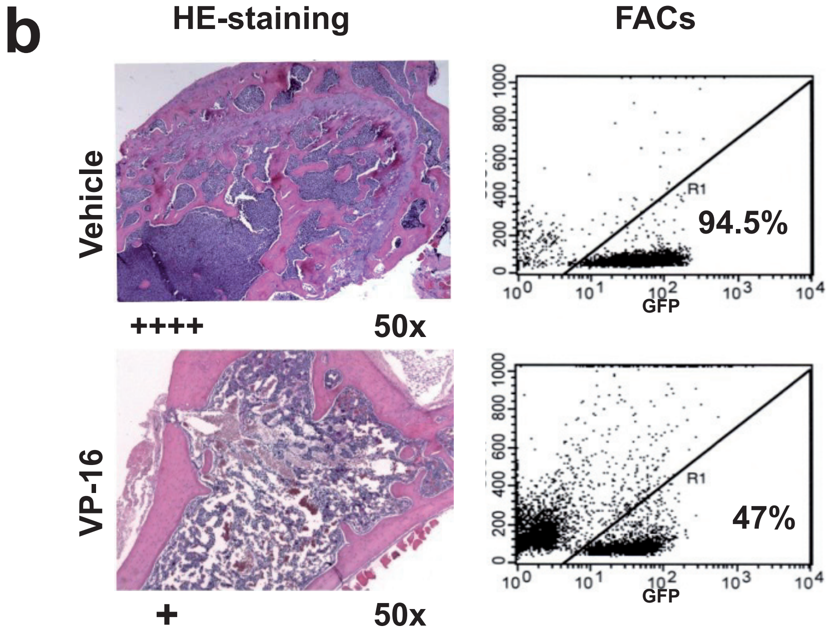
b
HE-staining
FACs
Vehicle
VP-16
++++
50x
+
50x
R1
94.5%
47%
GFP

c **ALL-199 + VP-16**

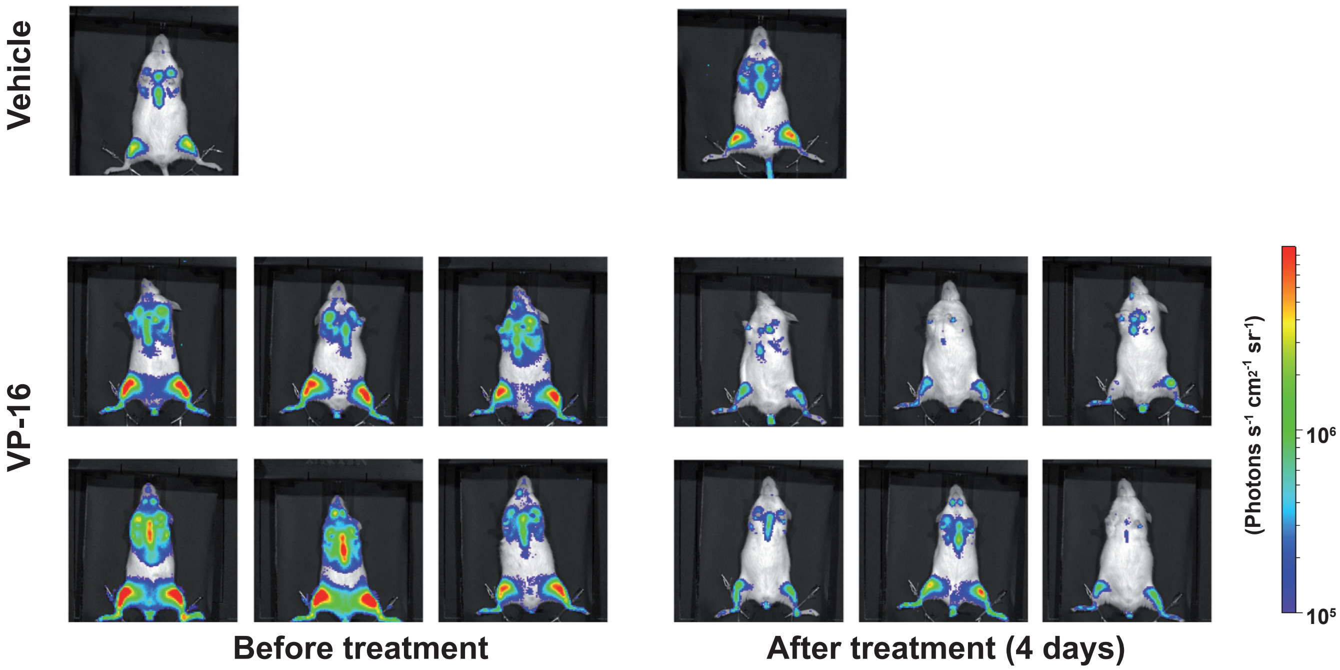

d

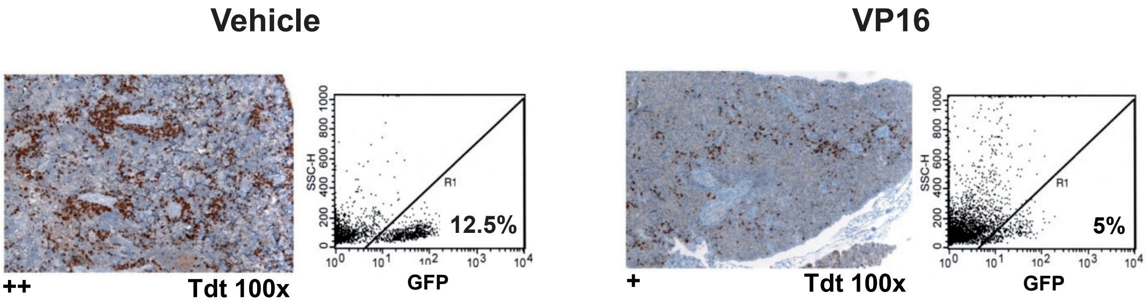

e

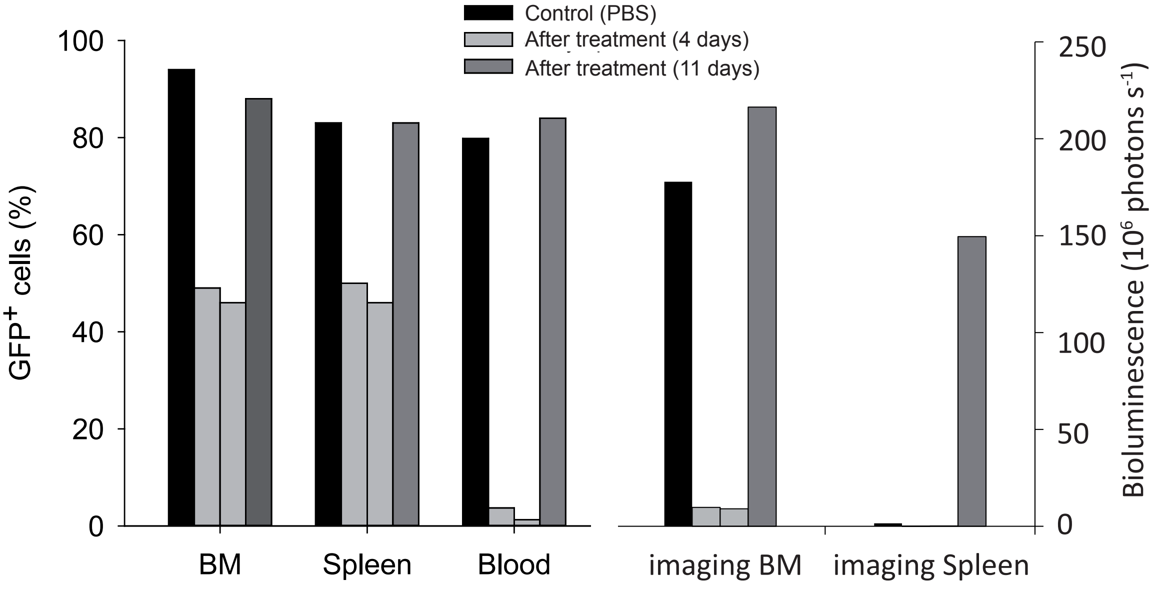

Suppl. Figure 11

Correlation of in vivo imaging to post mortem readout parameters for measuring treatment effects with low variances

10^5 ALL-50 (**a, b, e**) or 10^4 ALL-199 cells/mouse (**c, d**) were injected into 10 mice; one control mouse was treated with PBS, while the remaining mice received a single intraperitoneal dose of Etoposid (VP-16; 50 mg/kg) in week 6 post cell injection; (**a, c**) Animals were imaged directly before treatment and 4 days after treatment; (**b, d**) 1 mouse was sacrificed before and 1 mouse 4 days after treatment; bones (**b**) or spleens (**d**) were evaluated by flow cytometry for presence of GFP-positive cells and by immunohistochemistry (Tdt) for presence of leukemic cells; (**e**) The treatment effects and the follow up were evaluated by flow cytometry and in vivo imaging; 1 control mouse and 2 treated mice were sacrificed at day 4 after treatment and 1 treated mouse at day 11 after treatment and organs were analyzed for presence of GFP positive cells.

Sample		ALL subtype	Age at diagnosis (years)	Gender	1. disease / relapse	Leukocyte cell count	Cytogenetics and molecular genetics	Day of CR
**ALL-4S**		T	4	m	1.	573.000	46, XY, t(11;14);(p32;q11); rearrangement of the TAL1-gene with the T-cell receptor locus	33
**ALL-7S**		pre-B	0,4	m	1.	202.000	46; XY, t(2;15)(p13;q15)	40
**ALL-50**		pre-B	7	f	1.	unknown	unknown	unknown
**ALL-53**		pre-B	14	f	1. relapse	4.500	unknown	not achieved, death
**ALL-54**		pre-B	3	f	1.	59.600	negative for BCR/ABL, MLL/AF4, TEL/AML1	15
**ALL-169**		pre-B	18	f	1.	14.100	unknown	unknown
**ALL-177**		pre-B	8	f	1.	5.700	positive for TEL/AML1, deletion 12p	43
**ALL-188**		pre-B	9	f	1.	8.500	positive for TEL/AML1, negative for BCR/ABL and MLL/AF4	unknown
**ALL-199**		pre-B	8	f	2. relapse	3.800	unknown	not achieved, death

Suppl. Table 1 - Clinical data of ALL donor patients.
age at diagnosis = age, when 1. disease or relapse was diagnosed and when the sample was retrieved;

m = male; f = female;

leukocyte cell count = numbers of leukocytes in peripheral blood at diagnosis of 1. disease or relapse;

day of CR = day, when complete morphological remission in bone marrow was achieved.

mouse passage	GFP positive cells in the spleen (%)
**1**	90
**2**	92
**3**	87
**4**	88

Suppl. Table 2 - Stability of transgene expression over passaging.

Sample ALL-199 was transduced lentivirally to express GFP; GFP-positive cells were enriched by flow cytometry sorting once and re-passaged through mice; shown are the percentage of GFP-positive cells for 4 consecutive passages after enrichment as measured in cells isolated from the spleens of diseased mice.
